# Consumers’ perceptions of vape shops in Southern California: an analysis of online Yelp reviews

**DOI:** 10.1186/s12971-014-0022-7

**Published:** 2014-11-28

**Authors:** Steve Sussman, Robert Garcia, Tess Boley Cruz, Lourdes Baezconde-Garbanati, Mary Ann Pentz, Jennifer B Unger

**Affiliations:** Department of Preventive Medicine, University of Southern California, Los Angeles, CA 90032 USA; Departments of Preventive Medicine and Psychology, and School of Social Work, Institute for Health Promotion and Disease Prevention Research, University of Southern California, Soto Street Building, 2001 North Soto Street, Room 302A, Los Angeles, CA 90033 USA

**Keywords:** Vaping shops, Yelp, Southern California, Tobacco regulatory science

## Abstract

**Background:**

E-cigarettes are sold at many different types of retail establishments. A new type of shop has emerged, the vape shop, which specializes in sales of varied types of e-cigarettes. Vape shops allow users to sample several types. There are no empirical research articles on vape shops. Information is needed on consumers’ beliefs and behaviors about these shops, the range of products sold, marketing practices, and variation in shop characteristics by ethnic community and potential counter-marketing messages.

**Methods:**

This study is the first to investigate marketing characteristics of vape shops located in different ethnic neighborhoods in Los Angeles, by conducting a Yelp electronic search and content analysis of consumer reports on vape shops they have visited. The primary measure was Yelp reviews (N = 103 vape shops in the Los Angeles, California area), which were retrieved and content coded. We compared the attributes of vape shops representing four ethnic communities: African American, Hispanic/Latino, Korean, and White.

**Results:**

Vape shop attributes listed as most important were the selection of flavors or hardware (95%), fair prices (92%), and unique flavors or hardware (89%). Important staff marketing attributes included being friendly (99%), helpful/patient/respectful (97%), and knowledgeable/professional (95%). Over one-half of the shops were rated as clean (52%) and relaxed (61%). Relatively few of the reviews mentioned quitting smoking (32%) or safety of e-cigarettes (15%). The selection of flavors and hardware appeared relatively important in Korean ethnic location vape shops.

**Conclusions:**

Yelp reviews may influence potential consumers. As such, the present study’s focus on Yelp reviews addressed at least eight of the FDA’s Center for Tobacco Products’ priorities pertaining to marketing influences on consumer beliefs and behaviors. The findings suggest that there were several vape shop and product attributes that consumers considered important to disseminate to others through postings on Yelp. Lack of health warnings about these products may misrepresent their potential risk. The main influence variables were product variety and price. There was only a little evidence of influence of ethnic neighborhood; for example, regarding importance of flavors and hardware. Shop observational studies are recommended to discern safety factors across different ethnic neighborhoods.

**Electronic supplementary material:**

The online version of this article (doi:10.1186/s12971-014-0022-7) contains supplementary material, which is available to authorized users.

## Introduction

The popularity of electronic cigarettes (e-cigarettes) has increased much more rapidly than the research on their use, and potential benefits and/or harms for users and nonusers, and workers in retail environments that sell e-cigarettes. Scientific evidence is limited and filled with controversies regarding the overall harms versus potential benefits as a harm reduction or cessation tool when compared to combustible cigarettes [1]. Although many jurisdictions in the U.S. are increasingly restricting e-cigarette use in locations where combustible cigarettes are banned, and there are calls for greater regulation by the Food and Drug Administration (FDA), the reality is that e-cigarettes have piqued the interest of many potential users, including youth and young adults. Sales of e-cigarettes began in 2007, reached $1 billion dollars in 2013, and are becoming a potential source of growth of the market share among tobacco companies engaged in e-cigarette sales [2]. It is not yet clear what the impact of e-cigarettes will be on future use of regular cigarettes, social norms about smoking, and health. However, several concerns exist regarding potential negative effects while a subculture of use and perceived safety is rapidly developing [3–5]. Another concern is that the proliferation of e-cigarette use could exacerbate existing tobacco-related health disparities across racial and ethnic groups. Trial of e-cigarettes occurs more among White non-Hispanic adults than other ethnicities (6.8%), followed by Asian and other non-Hispanics (6.1%), African Americans (4.5%) and then Hispanics (3.9%) [2]. However, these patterns could change if e-cigarettes become more widely available in minority communities.

E-cigarettes are battery-powered devices that generally are used to vaporize nicotine (in various nicotine concentrations [and there are e-cigarettes without nicotine as well], generally in a propylene glycol [PG] and vegetable glycerin [VG] solution, also with flavoring). They may be disposable (e.g., 400 puffs of 24 mg to 30 mg nicotine for $7 to $9; popular brands include Blu Cigs and NJOY) or rechargeable (with battery chargers, heating coils, liquid solution; for $20 to $40; a popular starter kit is V2 Cigs). E-cigarette users (often called “vapers”) inhale a vapor that lacks many of the toxic compounds in cigarette smoke that contribute to the extremely high risk of disease and premature death in smokers. Given this promise of “harm reduction”, there is great interest in these devices. However, scientific understanding of the long-term consequences of their use, or the long-term impact on workers who breathe the “vapor” and handle the mixtures of nicotine flavored e-cigarette liquids (juices) all day, is quite incomplete at present. There are numerous toxins in e-cigarettes that vary across flavors and brands [1]. Of importance, recent work has indicated that 74% of e-juice sweet flavors include diacetyl and acetyl proprionyl, which are associated with respiratory diseases [6].

E-cigarettes can be purchased in tobacco retail environments, vape shops, and on the Internet [7]. Rather little is known regarding sales of e-cigarettes across types of retail establishments, but e-cigarettes are becoming ubiquitous in convenience stores, liquor stores, and pharmacies [8], and highly visible e-cigarette ads and self-service displays are present in nearly one-third of stores that sell e-cigarettes.

Stores devoted exclusively to sales and use of e-cigarettes are known as “vape shops”. There are at least 3500 vape shops in the U.S. [7,9]. Vape shops sell a variety of types of refillable and disposable e-cigarettes, several types of solution strengths and flavors, more complex and powerful tank systems that offer unique vaping experiences for experienced users, and sometimes other accessories (e.g., waterpipes). A Google search for “vape shops” with “Los Angeles” reveals over 5,000 pages (accessed 12-20-2013).The fact that the mark-up on e-cigarettes can be 200-400% (compared to 10-20% for combustible cigarettes) may account for the unprecedented growth in prevalence of such shops [9].

Although many believe e-cigarettes are much safer than combustible cigarettes, and some are very opposed to tobacco industry marketing tactics [10], vape shops might have a negative impact on health behaviors and health outcomes. If e-cigarettes are dangerous (e.g., nicotine itself may raise blood pressure, the products are heated which may make them carcinogenic, and the ingredients in flavorings may be toxic at certain dose levels and are not regulated), promotion of e-cigarettes may lead to ill health. Spills, inhalation of vapors, and lack of other safety precautions (e.g., not wearing gloves or goggles while handling juices, or liquid nicotine; or while drilling airholes or rebuilding) at these shops also may contribute to ill-health of workers and patrons. Also, to the extent that purchasers do not use e-cigarettes to quit regular cigarettes but instead use them to remain nicotine-dependent (dual use), or as a gateway to combustible cigarettes especially by youth and young adults, these shops may indirectly promote initiation and continued use of a variety of tobacco and nicotine products. More information about vape shops is needed to inform future regulations. Learning more about the physical environment of vape shops, as well as about the products sold, provides a ‘big picture’ of marketing strategies to reveal what may lead consumers to initiate and maintain a vaping habit. That is, the store layout, juice flavors, and hardware provides information about the ability of vape shops to entrench consumers in this social context and get them educated in a language and product line, and addiction to nicotine. Such etiologic information could inform the FDA’s development of future public information/education campaigns for specific groups of consumers [11].

Currently, there are no empirical research articles on vape shops. The present study uses a Yelp consumer review search to explore the vape shop environment. These reviews offer insights into the products and services provided by these shops, as well as the types of products, services, staff, and amenities that customers value. This study pertains to several Center for Tobacco Products, FDA Research Priorities (#s 1 [components of e-cigarettes], 3 [consumer use behavior], 4 [consumer attitudes and beliefs, marketing influences], 20 [perceived lowering of product toxicity and behavior], 39 [nature of discussions in social networking sites, subpopulation differences], 40 [communication channels vulnerable populations may use], 49 [price promotions impact], and 51 [vulnerable populations attitudes and beliefs]; see http://www.fda.gov/downloads/TobaccoProducts/NewsEvents/UCM293998.pdf; accessed 6-13-2014).

We hypothesized that the vape shop environment promotes an easily accessible means to try out the product (location and promotion), a language of e-cigarette use, and expectation that use is relatively safe. In addition, we examined the possibility that vape shops create and perpetuate a vaper identity that might be attractive, similar to the physical environment of “head shops” that sell marijuana accessories [12]. We also explored differences in vape shop characteristics across ethnic communities. Such information would be particularly useful to provide inputs on the creation of FDA public education campaigns and identification of misleading information that may be conveyed in these shops.

## Methods

### Data extraction: using data from Yelp

Yelp is a multinational corporation headquartered in San Francisco, which operates in large part as a business review site, within which customers may exchange business recommendations. The reviews involve a search engine that can locate specific types of businesses by neighborhoods/cities. Reviews permit updateable narrative description, and include a rating system (one to five stars, least to most favorable). Gender of reviewer can be determined in most cases, by name, photo, and narrative information provided by the reviewer. The company attempts to remove fake reviews (self-promotion), though some fraudulent reports may still occur [13]. Its use of an unweighted arithmetic rating system has been criticized as simplistic [14,15], and reviews could be influenced relatively more by frequent reviewers [16]. However, consumers primarily do use Yelp as an information source for products and services [17,18], and multiple reviews tend to become less extreme after a shop opens [15]. Currently, the company has a Better Business Bureau rating of A+ [17,18]. Analysis of Yelp reviews is a promising strategy to identify the characteristics of businesses that customers view as most salient in different locations [19], without imposing the researchers’ opinions about which characteristics should be most important to consumers.

### Location of Cities/Neighborhoods

This study focused on four ethnic communities: Korean, African American, Latino, and White. These communities were selected because they are important vulnerable populations for tobacco control efforts, and each of these racial/ethnic groups has a unique tobacco use profile. For example, Latinos are the largest minority group in California; African Americans have a characteristic late onset of tobacco use, a high prevalence of menthol tobacco use, and disproportionate rates of tobacco-related disease; Koreans are an Asian American subgroup with especially high tobacco use prevalence compared with other Asian groups; and Whites in the U.S. are more likely than other groups to use tobacco during adolescence and to use smokeless tobacco products compared to other ethnic groups [20–23]. These characteristics may make each of these communities an attractive target for vape shops. To identify neighborhoods/cities relatively high in Korean, African American, Latino, and White ethnicity, we used published analyses of U.S. Census data [24–26] to determine the racial/ethnic composition of the neighborhood surrounding each vape shop.

Using the names of neighborhoods/cities located (e.g., Koreatown, Exposition Park, Commerce, Hermosa Beach), Yelp was then searched using that neighborhood/city term. Only shops that were reviewed by at least five reviewers were coded; this resulted in a sample of 103 vape shops. Coders were instructed to code the most recent reviews, up to 20 of them for each shop, to try to capture the least biased reviews [15].

After at least 20 shops were located for an ethnicity, the search for that ethnicity ended. We included all shops with 5 to 20 reviews in all neighborhoods/cities located, such that we might cross over a 20 shop threshold (for ample statistical power to detect location differences [1-beta = .8], resulting in a slightly different number of shops per ethnic neighborhood. The final sample consisted of 22 vape shops in the most highly Korean neighborhoods in Los Angeles (ranging from 32% to 8% Korean), 30 vape shops in the most highly African American neighborhoods (ranging from 38% to 14% African American), 25 vape shops in the most highly Latino neighborhoods (ranging from 93% to 63% Latino), and 26 vape shops in the mostly highly White neighborhoods (ranging from 85% to 70% White).

### Calculation of prominence scores

Next, we attempted to verify that we had selected a sample that would be likely to be seen by customers searching on Google for vape shops. To do this we created a “prominence estimate” of using the Yelp web site to locate shops. We summed up the number of Yelp web sites on the first 10 web pages of Google (across the 45 locations that had been selected, mean = 5.42, SD = 2.47). Then, we added up the number of non-Yelp sites on the first 10 web pages for each location that mentioned a shop on the list of shops selected to be coded (that is, that had been selected by having at least 5 reviews in Yelp; mean = 1.53, SD = 1.38). Next, we combined the number of pages of either type (mean = 6.96, SD = 2.07 across the 45 locations). That is, 69.6% of the sites examined were either Yelp sites or otherwise included a selected vape shop for coding from that location. The remaining cases were those of alternative vape shop locating web sites (www.rankmyvape.com, http://vape.locate.com, www.e-cigarette-forum.com), the yellow pages, distributors of e-cigarettes but not shops, YouTube videos of e-cigarette use, other shops listed on Facebook, and other specific shop web sites. Thus, an apparently total prominence of vape shops was indicated.

### Developing the coding measure

In Yelp, pairing “vape shop” with “Near Los Angeles, CA” revealed 490 vape shops [19]. We sampled the third shop on every third page, up to the 15th page from this Los Angeles area search to provide examples of vape shops on which to develop a coding measure. We noted that comments made were about shop and staff characteristics viewed as important, the physical layout of the shop, suggestions that e-cigarettes were a safe alternative to smoking, the range of rechargeable products and parts, and the variety of liquids and mods (mechanical modifications used to increase vapor production or nicotine yield). A coding sheet was developed based on an initial qualitative analysis of the recurring themes in these Yelp pages.

### Coding measure

First, the measure indicated general information: the vape shop reviewed, gender of reviewer (male, female, not known), total number of reviews on site, number of reviews completed (ranged from 5 to 20 because shops with fewer than 5 reviews were excluded, and a maximum of 20 reviews were coded; as is described below), dates of earliest and most recent coded reviews, and vape shop information (name, phone address). Next, the measure indicated characteristics noted as important in this vape shop: never rushed by employees, wide range of nicotine in juices, great selection of flavors or hardware, unique flavors or hardware and examples of each, whether or not there were fair prices, on-line store capability, and rebuilds/fixes. The number of reviewers reporting each of these characteristics was determined. An “other characteristics” category also was included, which indicated whether reviewers made comments not fitting into one of the previous categories. Examples of other characteristics were open-ended coded.

Third, endorsements of staff attributes were coded in terms of person-to-person marketing influence characteristics such as helpful/patient/respectful, knowledgeable/professional, friendly, good personality (e.g., cool, relaxed), quick service, let customer try out lots of flavors, and other attributes (with open-ended coding). Fourth, the reviewers’ comments about marketing influences pertaining to the physical environmental layout level were coded. Coding of marketing physical environment pertained to venue type (bar, club, or “other” category such as lounge or head shop, the latter which was open-ended); venue amenities (good or bad parking, clean or not, types of furniture, lighting, art, music, presence of TVs, water tank, mugs/coffee, chalkboard menu, and other amenities, the last nine categories permitting open-ended coding as well as yes/no endorsement via number of reviewers), and marketing atmosphere (chic/classy, relaxed, fun, “awesome” [stated verbatim by reviewer], or other; all of which measured number of reviews that mentioned them as well permitting open-ended coding).

Fifth, health claims information was coded; that is, number of reviewers that stated that e-cigarettes are relatively safe; and one can quit smoking at that shop. Other health claims were also noted and open-ended coded. The coding sheet used is attached as Additional file 1.

### Intercoder agreement on measure

A second reviewer coded the reviews of 16 vape shops (randomly selected from a list of all 103 vape shops, stratified by ethnic community). All closed-ended categories were considered in the coding. Intercoder agreement for the two reviewers was calculated using Cohen’s Kappa [27]. Across 37 comparisons, agreement varied from almost perfect agreement (.81 or higher, 17 comparisons), substantial agreement (.61-.80, six comparisons), moderate agreement (.41-.60, four comparisons), fair agreement (.21-.40, seven comparisons), and slight agreement (.20 or lower, three comparisons). We discarded variables that had Kappas lower than .30. These included other characteristics, quick service, other physical environment attributes, bad parking, and furniture.

### Analysis

The unit of analysis in the present study was the vape shop. Chi-square tests were conducted to examine variation across ethnic communities in vape shop characteristics mentioned on Yelp (Fisher’s Exact Test was used for analyses with expected cell counts less than 5). The location variable was coded into categories based on the ethnic make-up of the community, as described above. The analysis included characteristics from the different coding measure categories: important shop characteristics, staff attributes, physical environment, atmosphere, and health claims. Prior to calculating intercoder agreement or performing the chi-square test, the shop characteristics were recoded into dichotomous variables, at the store level. If a characteristic had no mention in the store review it was coded with a value of 0 and if it was mentioned then the value was coded as 1. We examined through scatter plots whether or not a characteristic was relatively likely to be endorsed as a function of number of reviews coded, but failed to find such a relation. Thus, store-level coding appeared to be a reasonable means to gauge prevalence of different characteristics at stores as a function of ethnic location.

## Results

### General information

There were 1556 Yelp reviews represented in the coding of 103 shops (mean = 15.11 reviews per shop, SD = 5.08). These represented 47.54% of all reviews across these shops, including the older non-coded reviews (total of 3273; mean = 31.78, SD = 41.34; 41 of the shops had more than 20 reviews completed). Among the reviewers, 57% were male, 28% were female, and 15% were unknown. Also, 83.8% of the reviews were 5 stars, 5.7% were 4 stars, 2.4% were 3 stars, 2.8% were 2 stars and 5.3% were 1 star. Thus, most of the reviews were rather favorable and were completed by males. The reviews took place over an average 6.57 months (SD = 4.72 months), the most recent extending from 10-25-2013 to 5-16-2014. As described above, information was aggregated to the store level. That is, if a characteristic appeared once among the reviews at a shop, it was coded as present (or 1). Only if a characteristic did not appear in any of the reviews was it coded as absent (or 0). The specific shop characteristics, staff attributes, physical environment, atmosphere, and health claims results are shown in Table 1.

**Table 1:** **Characteristics of vape shops across ethnic communities**

	**All**	**Korean**	**Hispanic**	**White**	**African-American**
Characteristics important in this vape shop					
Great Flavor or Hardware Selection*	95.2	100.0	96.0	92.6	93.1
Fair Prices*	92.3	100.0	92.0	81.5	93.1
Unique Flavors or Hardware*	89.3	86.4	92.0	88.9	89.7
Rebuilds or Fixing	65.1	72.7	68.0	63.0	58.6
Never Rushed	26.2	27.3	32.0	25.9	20.1
On-line Store Capability*	7.8	13.6	4.0	0.0	13.8
Wide Range Nicotine*	5.8	13.6	4.0	3.7	3.5
Other Unique Qualities^1*^	76.7	95.5	52.0	81.5	79.3
Staff attributes mentioned					
Friendly	99.0	100.0	96.0	100.0	100.0
Helpful/Patient/ Respectful*	97.1	100.0	92.0	96.3	100.0
Knowledgeable/ Professional*	95.1	100.0	92.0	100.0	89.7
Good Personality (e.g., cool, relaxed)	79.6	86.4	68.0	81.5	82.7
Let Me Try Out Lots of Flavors	78.6	77.3	72.0	85.2	79.3
Quick Service*	12.6	13.6	0.0	14.8	20.7
Other Attributes^2^	99.0	95.5	100.0	100.0	100.0
Physical environment and amenities					
Clean	52.4	63.6	40.0	51.9	15.5
Bar Type*	39.8	22.7	36.0	63.0	34.5
Good Parking*	33.0	27.3	24.0	55.6	24.1
TVs	25.2	24.1	32.0	14.8	24.1
Art*	18.5	18.2	16.0	14.8	24.1
Lighting*	16.5	22.7	8.0	22.2	13.8
Club Type	0.1	0.0	4.0	0.0	0.0
Other Amenities^3^	87.4	95.5	80.0	96.3	79.3
Other Venue Type^3^	42.7	45.5	28.0	55.6	41.4
Atmosphere					
Relaxed	61.2	50.0	68.0	59.3	65.5
Awesome*	12.6	13.6	8.0	11.1	17.2
Chic/Classy*	6.8	4.6	4.0	11.1	6.9
Fun*	5.8	9.1	0.0	7.4	6.9
Other^4^	67.9	72.7	52.0	77.8	68.9
Health claims					
Can Quit Smoking Here	32.0	36.4	28.0	33.3	36.4
E-cigarettes are Safe	14.6	4.6	12.0	33.3	6.9

Notes.

^1^Other qualities include rewards program or discounts or warranty, own house blend, offer drilling and torching, can adjust nicotine dosage, allows pets in shop, great hours, raffles, and can drip juices to test.

^2^Other attributes include non-judgmental, comedic, honesty, overall great customer service, caring, treated like fellow vape geeks, and accommodating.

^3^Other venue types include lounge with art, simple and organized. Other amenities include music, water tank, mugs, chalkboard e-juice menu, great location, gaming, chess/checkers, pool table, food, and coffee.

^4^Other lifestyle environment includes: friendly, feel good vibes.

*Significant at the p <0.05 level for chi-square test for independence or Fisher’s exact test.

### Characteristics noted as important and staff attributes mentioned

Overall, the vape shop characteristics mentioned most frequently were great flavors or hardware selection (95%), fair prices (92%), and unique flavors or hardware (89%). The endorsement of these marketing characteristics varied significantly across ethnic communities. Great flavors/hardware selection and fair prices were mentioned most often for stores in Korean communities and least often for stores in White communities (Fisher’s Exact p = 0.04 & p < 0.01). Having unique flavors/hardware were mentioned most often for stores in Hispanic communities and least for stores in Korean communities (Fisher’s Exact p = 0.03). Other characteristics that varied significantly across ethnic communities were online store capability (8% overall; mentioned most often for stores located in African American and Korean neighborhoods and least often for stores located in White neighborhoods; (Fisher’s Exact p < 0.01) and wide range of nicotine (6% overall; mentioned most often for Korean stores and least often for African American stores; Fisher’s Exact p = 0.02).

The staff attributes mentioned in vape shops (regardless of ethnicity location) most frequently were friendly (99%), helpful/patient/respectful (97%), and knowledgeable/professional (95%). Descriptions of staff as helpful/patient/respectful and knowledgeable/professional were significantly less common in Hispanic stores than in other stores (Fisher’s exact; p = 0.05 & p = 0.01), although over 90% of all stores in all ethnic communities were described as having helpful/patient/respectful and knowledgeable/professional staff. Good personality and “let me try out lots of flavors” were mentioned slightly less frequently (80% and 79%, respectively) and did not vary across ethnic communities.

### Physical marketing environment suggested

Although physical marketing environment characteristics were mentioned less frequently than store characteristics or staff attributes, physical characteristics such as cleanliness (52%), bar-type atmosphere (40%), and good parking (33%) were mentioned as descriptive of at least one-third of the stores. Physical marketing environment characteristics that varied significantly across ethnic communities were bar-type environment, good parking, art, and lighting. Bar-type environment was mentioned most frequently for stores in White communities (χ^2^ (3, N = 103) = 9.21, p = 0.03). Good parking was mentioned most often for stores in White communities ((χ^2^ (3, N = 103) = 8.48, p = 0.04). Having art displayed was mentioned most often for stores in African American communities (Fisher’s exact p = 0.01) while store lighting was mentioned most frequently for stores in Korean communities (Fisher’s exact p < 0.01). Other physical environment characteristics mentioned occasionally included friendly, feel good vibes, and relaxed. Very few reviews suggested that the shop’s atmosphere was chic/classy, fun or awesome. However, White community shops were more likely than other shops to be described as “chic/classy” (Fisher’s exact p = 0.03); African American community shops were more likely than other shops to be described as “awesome” (Fisher’s exact p = 0.02); and shops in non-Hispanic communities were more likely than shops in Hispanic communities to be described as “fun” (Fisher’s exact p = .0.02).

### Health claims

Relatively few stores received comments about health claims; 32% of the stores had reviewers who mentioned that the store was a place to quit smoking, and only 15% of the stores had reviewers who claimed that e-cigarettes are safe. These characteristics did not vary significantly across ethnic communities.

## Discussion

Importance of great flavors and hardware selection were the most prevalent characteristics reviewers identified in the shops (regardless of ethnicity location), which is not surprising as these are the featured objects provided within vape shops [28]. Person-to-person marketing influences tended to operate across all vape shops. As examples, staff members were frequently described as friendly, helpful/patient/respectful, and knowledgeable/professional across ethnic communities. There were more similarities than differences in vape shops across ethnic communities. However, some differences did emerge. Reviews of vape shops in Korean neighborhoods were relatively more likely to mention great flavors and hardware selection, fair prices, online store capability, wide range of nicotine, helpful/patient/respectful staff, knowledgeable/professional staff, and fun atmosphere. Reviews of vape shops in Hispanic neighborhoods were relatively more likely to mention unique flavors or hardware and relatively less likely to mention helpful/patient/respectful staff, and awesome, chic/classy, or fun environment. Reviews of vape shops in White neighborhoods were relatively more likely to mention knowledgeable/professional staff, bar-type atmosphere, and chic/classy atmosphere and relatively less likely to mention great flavor or hardware selection, fair prices, and online store capability. Reviews of vape shops in African American neighborhoods were relatively more likely to mention online store capability, helpful/patient/respectful staff, and awesome atmosphere. One may speculate that vape shop physical environment characteristics that appeal to different segments of consumers will keep these consumers in the shop for a longer period, facilitate instruction on hardware and use, and lead to initial or maintenance of addiction to nicotine.

Possibly contrary to some work on e-cigarettes [29], relatively few reviews mentioned health safety or quitting cigarettes as being intrinsic to the vape shop function. We did not detect a head-shop, medical marijuana, or alcohol related context through these reviews. That is, assuming the reviews are valid, it would appear that vape shops in general do not represent environments that actively promote public health (e.g., quitting combustible cigarette smoking) or encourage other risky behaviors (e.g., such as marijuana use). These are shops that focus specifically on the promotion of e-cigarette use as an activity of enjoyment and relaxation.

If one were to speculate on ethnic differences, possibly socioeconomic considerations might differentiate reviews, in terms of relative importance of price and décor of the shops, and possible types of hardware available at the shops. As Additional file 2 reveals, a great number of terms have developed to describe flavors and hardware at vape shops. It is possible that there is a general subculture around vaping and vape shops. It is likely that many of these shops are locations where customers would be permitted to spend time, vape, and engage in some other activities. This is significant because if vape shops also function as “hangout” locations, spending time in a vape shop might cause patrons to befriend other patrons, form more pro-vaping attitudes, initiate use of more powerful and expensive products, and increase their nicotine intake. Vape shops are places that permit the initiation, use, and maintenance of hardware to continue to use e-cigarettes with a better “draw” and lasting power (e.g., see Kim, Flaherty, & Clark [10]).

The present Yelp review was the first of a two-stage study on vape shops, used to develop our sample and refine measurement of the vape shop environment. In the second stage, we will travel to the shops that were reviewed by Yelp, interview store personnel, and observe products available at the shops. We will be able to examine convergent validity with these reviews, and better learn about potential ethnicity location differences and environmental influences on e-cigarette use within and nearby vape shops.

### Limitations and future research directions

As mentioned in the Introduction, use of Yelp is not without limitations. Yelp is capturing e-cigarette consumers who are already motivated to vape. This may explain in part why reviews tended to be rather positive overall. This study did not directly compare negative with positive reviews, though perhaps another research design that preselected positively and negatively (e.g., five star versus one star) reviewed shops could be considered in future work. The shops are located in areas that are relatively high in a particular ethnicity. This arrangement has its limitations as a means of studying ethnic variations pertaining to vape shops. We do not know the ethnicity of the employees or customers. For the Korean and African American locations, the percentage of those ethnicities is not a majority in those areas, only relatively high compared to other areas in southern California. Future research should involve observations of these shops and products, and interviewing employees, to learn more about this environmental context.

These data do not provide much data for potential regulation of vape shops; that would stem from traveling to the vape shops and engaging in the interviews. These data do provide what we believe are rich data that may be used to inform FDA public education campaigns. We suggest that messages they might design could focus on caution regarding flavor and hardware selection, potential harms on health, and raising awareness of physical environmental-level marketing influences that the e-cigarette industry employs -- that give potential consumers the perception that e-cigarette use is somehow relatively less harmful and addictive, or more fun. Just as health education and media literacy campaigns were able to teach the public that the tobacco industry exists to make money from smokers and is not necessarily a credible source of health information, perhaps health education messages about vape shops could emphasize that the fun, friendly vape shop owner is also a salesperson rather than a health expert.

## Conclusions

To our knowledge, this is the first social marketing study in health behavior research to use Yelp as a guide and, as such, is quite novel. This study helps to advance our scientific knowledge of the characteristics and valued qualities of these increasingly popular retail environments. This information likely will help inform future FDA public awareness campaigns should they pertain to these or other shops specializing in e-cigarettes, particularly on how to tailor the visual material and language being communicated. The FDA could also use this information to help identify vape shop characteristics that might be enticing to youth, and consider regulating youth access or youth-oriented advertising in the future. Science on e-cigarettes has not reached a threshold of certainty regarding their potential to do no harm [30]. Nevertheless, given the potential promise of harm reduction of e-cigarettes and their increasing demand, there is a need to regulate the sales of e-cigarettes and to examine further the vape shop retail environment in which they are assembled and sold. Many jurisdictions throughout the United States and at the federal level via the FDA are considering regulations that protect workers in vape shops, consumers, and the overall public’s health through control of ingredients and chemicals that may be found in e-cigarettes and in the mixing and handling of nicotine related products. Until evidence reaches a high threshold of certainty that these products will do no harm, further studies are necessary. Counter-marketing messages to reduce misinformation might be considered, regulations that prohibit sales of e-cigarettes to minors should be uniformly enforced, and protection of vape shop workers from the any harmful effects of these products should be maximized.

## Additional files

Additional file 1:
**Yelp Vape Shop Review Measure: Coding Sheet Used for Yelp Vape Shops reviews.**
Additional file 2:
**Examples of Flavors and Hardware Mentioned at the Shops.**
