# Supplementary material for: Consumers’ perceptions of vape shops in Southern California: an analysis of online Yelp reviews
Source: Tob Induc Dis. 2014 Nov 28;12(1):22. doi: 10.1186/s12971-014-0022-7 (PMC4258055; doi:10.1186/s12971-014-0022-7)
Supplement: Additional file 1: — Yelp Vape Shop Review Measure: Coding Sheet Used for Yelp Vape Shops reviews. [file 12971_2014_22_MOESM1_ESM.pdf]

Appendix 1: Yelp Vape Shop Review Measure
(Store#______)
Date Collector: ___________________________ Date:_______________
Web site: ____ Reviewer Gender: #___M #___F #___IDK
Total number of reviews on site: _____ in English (#___5 star, #___4 star, #___3 star, #___2 star, #___1 star)
Number of reviews completed (most recent 5-to-20) ______ Dates of reviews:__/__/__-__/__/__
Vape shop name: ____________________________________ Phone: (____)

Address/City/State/Zip: ____
Location:_______________________

---

1. **Characteristics noted as important in this vape shop:**
   Never rushed #____ Fair prices #____
   Rebuilds/rebuilt/fix # _____
   Wide range of nicotine #____ On-line store capability #____
   Great selection of flavors (juices) or hardware #____
   Unique flavors or hardware #____
      Examples of unique flavors:__________________________________
      Examples of unique hardware:________________________________
   Other #_____
   Describe:____

2. **Staff attributes mentioned (e.g., friendly, helpful):**
   Helpful/patient/respectful #____
   Knowledgeable/professional #____
   Friendly #____
   Good personality (e.g., cool, relaxed) #____
   Quick service #____
   Let me try out lots of flavors #____
   Other #_____
   Describe:____

3. **Lifestyle environment suggested (e.g., alternative, relaxed, pleasurable, freaky, ethnic):**
   Venue Type
   Bar type: Yes#____No#____ (e.g., wooden bar for tasting)
   Club: Yes#____No#____ (e.g., TV's & lighting)
   Other: Yes#____Describe:____
   Venue Amenities
   Parking: Good #____ Bad #____
   Clean: Yes #____ No #____
   Furniture: Yes #____ Describe:____________________________________ (e.g., modern couches)

Lighting: Yes #____ Describe:________________________________ (e.g., bright LCD lights)
Art: Yes #____ Describe:_________________________________ (e.g., gnarly paintings)
Music: Yes #____ Describe:_____________________________________ (e.g., great music)
TVs: Yes #____ Describe:_____________________________________
Water tank: Yes #____ Describe:_____________________________________
Mugs: Yes #____ Describe:______________________________ (e.g., for coffee/hot cocoa)
Chalkboard menu:#____ Describe:_____________________________________
Other Yes #____ Describe:_____________________________________

Atmosphere

Chic/classy atmosphere: Yes #____
Describe:_____________________________________
Relaxed atmosphere: Yes #____
Describe:_____________________________________
Fun atmosphere: Yes #____
Describe:_____________________________________
Awesome atmosphere: Yes #____
Describe:_____________________________________
Other: Yes #____
Describe:_____________________________ (e.g., freaky)

4. **Health claims:**
E-cigarettes are vapor and safe #_____
Describe:____
Can quit smoking here #_____
Describe:____
Other #_____
Describe:____

5. **Any negative comments:**
Limited number of flavors # _____
Other #_____
Describe:____

6. **Ethnic or gender-specific comments:**
Hardware for girls/guys #_____
Describe:____
Other #____
Describe:____

7. **Other comments:**
Please list any notes or other comments:
____
____
