# Supplementary material for: Consumers’ perceptions of vape shops in Southern California: an analysis of online Yelp reviews
Source: Tob Induc Dis. 2014 Nov 28;12(1):22. doi: 10.1186/s12971-014-0022-7 (PMC4258055; doi:10.1186/s12971-014-0022-7)
Supplement: Additional file 2: — Examples of Flavors and Hardware Mentioned at the Shops. [file 12971_2014_22_MOESM2_ESM.pdf]

Appendix 2. Examples of Flavors and Hardware Mentioned at the Shops

Flavors: Island Bliss; Pink Starburst; Blueberry Mojito; Tropic Freeze; Fireball; Apple Cinnamon; lychee; ube; Hollywood Frost; Space Jam; Pluto; Kona Coffee; rocket pop; treasury; pink starburst; lemon heads; VLS (brand); crème de melon; berry hibiscus; deux menthe; dragon spit; supreme steam mother pucker; Venom; antidote; high roller; John Wayne; Vanilla Milkshake; Mango mixes; fruit flavors; drink flavors; Ruthless brand juices; Big Stick; Black Label Belgian breakfast; Johnny Chimpo; gummy bear; apple jacks; lychee; ubus; ice man; Honey Do; pink starburst; Colombiana; Azul juice; Black Magic; pine coolaid; LA cheesecake; Adam's Apple; Pina Dew Lada; vanilla custard; white girl; Diwata; Cool Apple; Sneaky Peach; Monster; Blue Hawaiian; Lola's Guava; pink starburst; peaches and strawberries; Cancun Ice; Pink Starburst; Chillaxin; Bonnie and Clyde; Astro; Planet of the Grapes; Bumble Bee; Panty Dropper; Choco strawberry; Mother's Milk; bamskillicious; STFU; Free Mason Elixir; E-Liq Cube; Double Down; Island Bliss; Grape Sac; Bluffing; Gummy Bears; RY4; Cool Watermelon; calamansi; Ube coconut; peanut butter; nostalgic candy; Kiss My Vape Apple; Beast Energy; Bull Range; Boden's Mate; Nectar; Mango Mist; Peach Breeze; Danish Caramel; Blueberry Custard; cucumber mint; Space Jam; triple C juice; Blue Waffle; Chi Chi; Monkey Biz; Big Melons; Unicorn Milk; Captain Crunch; Water Dew; Pink Bunny; Mr. J's; Horchata; Monkey Breath; The Lava; Scoudrel; Banana Blitz; Bumble B; Pink Spot; Justice; Chai organic; peppermint bark; throwback; the godfather; melon/menthol; green apple/peach; cosmic frog; kryptonite; milk n' honey; 24/7; Maja Blanca; peanut butter, nostalgic candy; Alice in Vapeland; cherry; craft; mother’s milk, unicorn blood, the dude; suicide bunny; nice juice; blueprint; five pawns; Dawn of Truth; Harlequin (juice); youngberry crunch; Rip Tide; Melon Breeze; Jamaica Me Crazy; pomegranate champagne; Free Mason Elixir; Shockwave; Dr. Pepper; mixed mint w/

chocolate; time bomb; Arctic Wolf; strawberry obsession; plum crazy; XTS; French Toast; Pamplona; blueberry cheesecake; sour skittles; Lucky 22; five pawns; Jamison; Honey Bager; SAC SAC; MSG juice; Crusty Custard; A-La-Mode; custom made tobacco flavor; Taro Coconut Cream; bluffin; ginseng chocolate mint; menthol; main squeeze; Nonne Torta Di Limone; Mash-Up; Abba bomb; Mystic mist; Chimp Berry; Monkey Cream; Apple all day; mama's cookie; doc drew; boba tea; monkey beach; Brain Freeze; Double Down; Mango Zone; watermelon; puma bomb; H20 melon; iced tea; Hurricane Malyssa; backyard boogie; vape blend #2; Doomsday; Cran'Chi; Dragon'Chi; Razz apple; manic melon; Hawaiian crown; vanilla sunrise; strawberry mango freeze; 4YR; #dr. greenthumb; pixi stix; sinigang

Hardware: steam turbine; Kayfun Lite Plus; provari mini; gold plated turtleship Chi-you mod; rhodium piece w/ rose gold rings; Dragon build; hammer mod w/ aqua; RDA caps; Kayfun; kind; nemesis magnets; VV; Spartan mechanical mod; EVOD clearomizers; Stingray mod; Ovale; pass-through; black chi you clone; Ego twist; clones; mods; dual coil atomizer; carton tanks; mod and atty; telescopic mod; dripping tank; K101; atty; Clone Atty Drip tip; 18650 battery; RBTA; iSmoka BCC Mega; hybrids; immortalizer; rsst; Protank; mech mods; 2.8 OHM Vivi Nova; nimbus; silica; iGoL; Deus Mods Prism V1; Taste Model; ego gen starter; Pyrex chroma tanks; itaste VV; ego-v; iSmoka tanks; Blow Pen; electric vapor hookahs; K100; IGO-W; gold drip tip; Survic Rat tanks; Joyten Twist 650; Krangertech protank 2; Segelei Telescopic Mod; K100; Steamboy; vaporizer w/ a dry flower attachment; RDA IGO W UD; cotton build; dragon; Kraken rebuildable in gold; Vape Queen two toned Colossus Mod; K100; VV mod; Pinoy mods; genesis; vision spinner 1300mah; ceramic wicks; joytech passthrough; Smoketech 3.5ml DCT; locking carotmizer; K100 dripper; apv mechanical mods; own mod production

company; special rare mechanical mods; originmod w/ aqua RBA; Titan of the Sea; matte blue e Go C Twist battery; Kangertech aero tech; I clear 30; igow3; Pax Ploom; Nemesis 3.1 Kayfun; clone attys; Aqua; isotope mod; Kangertech EVOD tank; Joytech Ego C Twist; Nemesis clone mod; VV/VW mod; MVP 2; Kanger ego Pro tank 2; Kayfun Lite; Nemesis Russian rebuild; Itazte 134; Magneto; Kayfun clone tank; Panasonic battery; Wool sleeves for SS cable; Paragon mod; Patriot dripper.
